# Talent management in volatility, uncertainty, complexity, and ambiguity (VUCA) health environment, nurses’ psychological contract fulfillment, cordial relation and generation: moderation-mediation model

**DOI:** 10.1186/s12912-024-02506-7

**Published:** 2024-12-03

**Authors:** Nancy Sabry Ellethiey, Heba Mohamed Al-Anwer Ali Ashour, Nadia Hassan Ali Awad

**Affiliations:** 1 Nursing Administration Department, Faculty of Nursing, Alexandria University, Alexandria, Egypt; 2 Present Address: Faculty of Nursing, 9 Edmon Fremon St. Smouha, Alexandria, Egypt

**Keywords:** Talent management, Generation, Psychological contract, Cordial relation, Nurses, Mediating-moderating model

## Abstract

**Background:**

The VUCA in the healthcare environment requires combating volatility, uncertainty, complexity, and ambiguity through highly talented employees and implementing a talent management strategy. This encompasses a range of operations designed to find, attract, nurture, and utilize competent workers that impact how well nurses fulfill their psychological contracts. However, as the researchers had anticipated, several factors, such as cordial relationships and generation differences, may mediate or influence this correlation.

**Methods:**

For a cross-sectional study, a non-probability convenience sampling technique was used to include *n* = 375 nurses from among the 600 total nurses at three private hospitals in Alexandria, Egypt. Three validated measures were used to measure the study variables and develop a mediation-moderation structural equation model.

**Results:**

The result of this study revealed that nurses perceived a moderate level of talent management with a mean score of (48.91 ± 18.15), a low mean score (8.89 ± 3.93) of psychological contract fulfillment, and a moderate mean score (185.11 ± 27.02) of cordial relation. Additionally, more than half of the participants were in the Gen Z. Also, this study concluded that there is a positive mediation role of cordial relation and a negative moderation role of Gen between talent management and psychological contract fulfillment.

**Conclusions:**

Using Gen as a moderating variable and cordial relations as a mediating factor, a moderating mediating structural equation model is created and validates the important influence of talent management on nurses’ psychological fulfillment, confirming the mediating effect of cordial relations and the moderating effect of genes in this relationship. GEN negatively predicted psychological contract fulfillment, meaning baby boomers predicted more psychological contract fulfillment than Z gen. Furthermore, talent management could positively predict cordial relations and psychological contract fulfillment, and cordial relations partially mediated the relationship between talent management and psychological contract fulfillment. Nurse mangers should be aware of and implement effective and talent management strategies with respecting gen difference, and apply tailored strategies for fostering nurses’ cordial relation and psychological contract fulfillment to deal with the VUCA challenging healthcare environment.

**Supplementary Information:**

The online version contains supplementary material available at 10.1186/s12912-024-02506-7.

## Background

Talent management (TM) is gaining remarkable recognition in the healthcare organizations nowadays since it the way for its future success in the market. Therefore, it is essential for employers not only to attract but also to develop and retain critical talent and to ensure that all the employees are highly engaged to improve organizational productivity [1]. Several studies explained a positive relationship between talent management and psychological contract fulfillment (PCF) which is considered the foundation for human resource management and acts as a psychological link between nurses and their managers [2, 3]. However, a question that comes to the researchers’ mind is whether the relationship between talent management practices and the psychological contract will be the same for all employees from different generations or whether this relationship may be affected by employee-employer cordial relations. Therefore, several elements could influence this relationship between talent management and psychological contract fulfillment, including gen as a moderating factor or cordial relation as a mediating component, especially in a VUCA environment in which nurses’ work that is characterized by Volatile, Uncertain, Complex, and Ambiguous circumstances. All these questions are what the researchers strive to answer and build their research framework [4–7].

## Theoretical framework

One may argue that by using talent management strategies, the organization is sending signals to its staff members following the signaling theory. These signals offer insights into potential daily experiences within the organization. Also, Social Exchange Theory (SET) that is based on the interchangeability process between the employer and employees. From this vantage point, it is possible to argue that talent management procedures influence employees’ perceptions of the organization and how it treats its bright workers, and psychological contract fulfillment is achieved by this reciprocal expectation of what the business expects from its personnel and what they can expect from it. This relationship may be affected by other contextual factors such as employee-employer cordial relations and generational differences. A generation is a cohort of individuals who belong to the same age group and have undergone or will undergo similar life experiences that have shaped their personalities. Consequently, different generations can be distinguished based on birth location and date, upbringing, educational patterns, and cultural and social environments. As a result of growing up in the same macro environment, individuals belonging to a particular generation tend to exhibit a shared set of behaviors, attitudes, and perspectives [8–10].

**Fig. 1:**
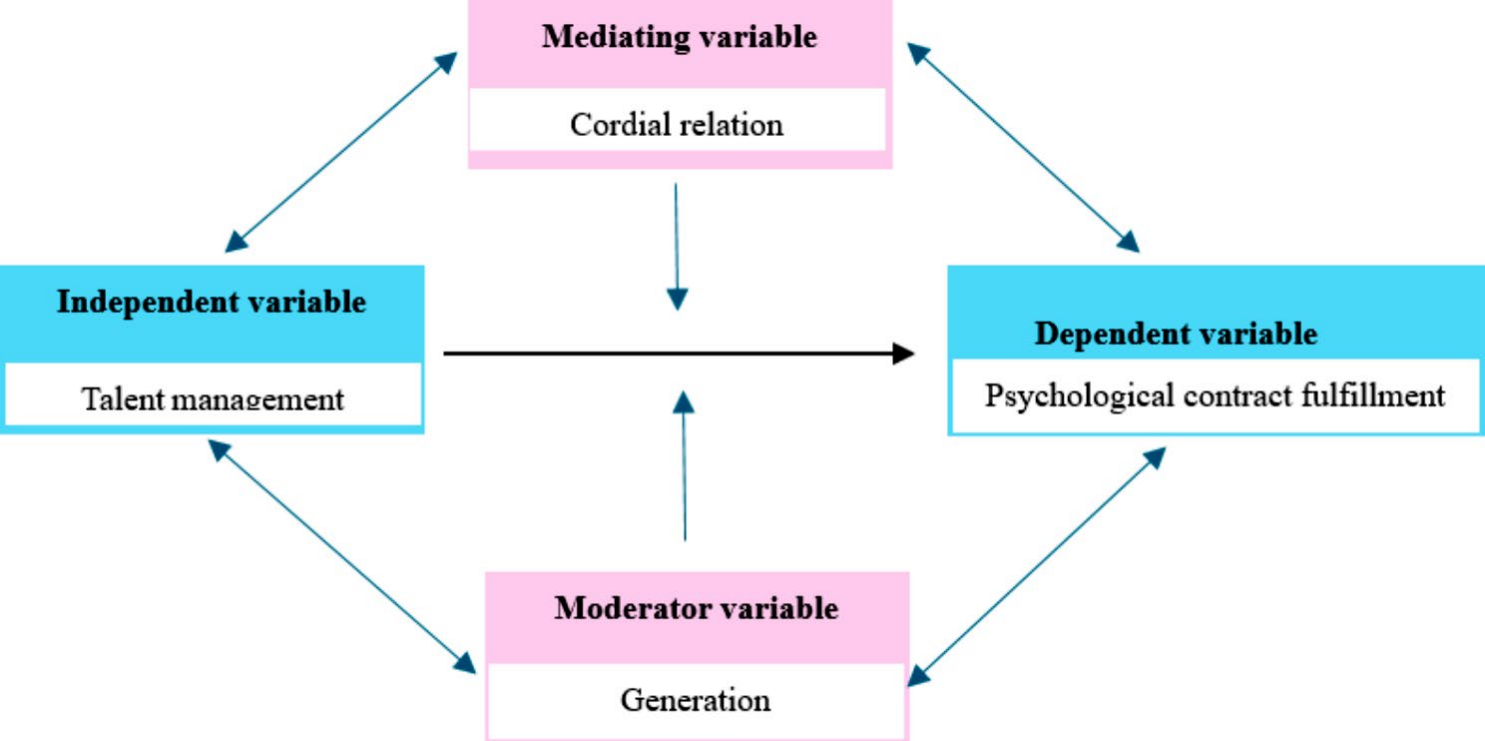
Proposed researchers’ conceptual framework

Additionally, the KAB model and ABC theory support the researchers’ framework. Knowledge, Attitude, Behavior model (KAB) developed by Kallgren and Woods (1986), and posits a sequential flow where knowledge acquisition leads to the formation of attitudes and drives behaviors [8]. The Attitude–Behavior–Context (ABC) theory, proposed by Guagnano et al. (1995) provides a more nuanced understanding of the attitude-behavior relationship by incorporating contextual factors. This theory suggests that behavior is the interplay between attitudes and contextual factors [9]. These assumptions are reflected in the study hypothesis that hospitals can employ strategies like talent management and education companies that enhance effective nurses’ knowledge toward managers’ initiatives in talent management to heighten nurses’ environmental concerns while shaping favorable attitudes towards organizations that form nurses’ psychological contract fulfillment. In this study, Gen and cordial relations act as contextual factors that shape nurses’ behaviors toward the organization. The KAB model and the ABC theory provide valuable insights and highlight different aspects of the attitude-behavior gap. The KAB model is instrumental in understanding the role of knowledge and attitudes, whereas the ABC theory underscores the importance of contextual factors [8–10] (See Fig. 1).

## Talent management (TM)

Healthcare organizations now face contemporary issues including population aging, technological advancement, healthcare globalization and internationalization, decreased disposable income, and a shortage of skilled workers. Additionally, there is a significant international movement of high-performing healthcare experts and workers from various healthcare industries. To address these issues that face the healthcare industry, healthcare providers must implement talent management strategies and integrated talent management systems in response to the shortage of high-performance personnel [11, 12]. Talent management (TM) is the “systematic attraction, identification, development, engagement, retention, and deployment of those individuals who are of value to an organization, either because they are fulfilling business/operation-critical roles or because they have ‘high potential’ for the future” [13].

International research contends that putting integrated talent management systems in place will increase organizational effectiveness, lower production costs, and lower the risks to patient safety. Attracting, nurturing, and retaining exceptional employees have shown to be difficult, complex, and demanding undertakings in an environment as complex as the ones mentioned above [14–16]. Talent management was categorized into four categories by Tiwari and Shrivastava (2013): Growth, learning, and opportunities (Talent development), Compensation and Benefits (Reward and Recognition), Work Environment and Policies (Talent Policy), and Management Support (Leadership Support) [17].

In 2023 a study was conducted and emphasizing the critical role of effective talent management practices in enhancing employee productivity and overall organizational success [18]. TM practices can be seen as an investment in a reliable and durable collaborative relationship. As a result, an organization’s investment in TM approaches leads to heightened emotional involvement and a mutually beneficial relationship between the talent and the employee. TM practices mirror psychological contract fulfillment (PCF) through communicating to intelligent workers that their employers value them and are trying to meet their expectations. According to scholarly interpretations of social exchange theory (SET), TM measures how willing a company is to engage in skilled labor, which impacts PCF. Therefore, when gifted individuals feel that their company is fulfilling its obligations and commitments, they perform better [5].

## Psychological contract fulfillment (PCF)

The study of psychological contracts has drawn more attention over the years because it offers a distinct framework for understanding changes in employment relationships and work-related outcomes. The term “psychological contract” which has its root in social exchange theory (SET), describes people’s conceptions of the duties and responsibilities that employers and employees have to one another [19–21]. According to Karani et al. (2021), a “psychological contract” is a set of employee ideas about the reciprocal duties and responsibilities that exist between individuals and the company, with a focus on a bilateral exchange relationship [21].

Psychological contract fulfillment (PCF) refers to nurses’ understanding and perception of the mutual obligations that hospitals and nurses must perform, including the obligations owed by the hospital to the nurse and the nurse to the hospital. These obligations could include incentives for good work, chances for advancement, provision of training, and a certain amount of responsibility. PCF focuses more on the psychological demands and expectations of nurses; therefore, lowering nurse turnover requires examining nurses’ perceptions, expectations, and attitudes regarding the hospital-nurse relationship [4]. Psychological contracts significantly influence employee behavior and organizational outcomes, including both nonstandard services and employee performance. By shaping how employees engage with their work and the organization at large, psychological contracts are pivotal in driving engagement, knowledge sharing behaviors, and, ultimately, organizational effectiveness and performance enhancement [22]. It also includes making self-commitment pledges to a future transaction that is necessary to keep the employer-employee relationship intact [23]. One of the key factors influencing employee behavior is the fulfillment of a psychological contract. When the psychological contract is better fulfilled in the context of the employer-employee relationship, employee performance is positively impacted, which may indicate a cordial relationship between the two parties [24].

## Cordial relation as a mediator

The effectiveness of any organization is largely dependent on its human resources. Without the consistent and steadfast support of the workforce, no business can endure over the long term. Workplace relationships are essential to the psychosocial work environment and are a source of well-being for all employees. For sustainable success, healthcare organizations should concentrate on and preserve friendly ties with their personnel. Employee trust, manager commitment, and employer concern are the hallmarks of this friendly working relationship [25]. According to social exchange theory, the manager-employee relationship is the commitment between an employee and their employer or organization, and this connection is reciprocal and interchangeable. Therefore, when managers show their commitment to their staff, the staff members respond similarly, experiencing a sense of attachment to the company and taking pleasure in their membership [26].

Nursing managers should never lose sight of the essential component of caring. The health and well-being of staff nurses are equally dependent on the compassionate actions of nurse supervisors. Therefore, compassionate behaviors by nurse managers foster relationships with staff nurses and healthcare teams to progress healthcare [27]. Mutual problem-solving, attentive reassurance, human respect, an encouraging demeanor, an understanding of the situation’s special significance, creating a healing environment, fulfilling fundamental human needs, and affiliation needs are all examples of caring behavior [28]. In this way, the manager’s sensitivity and thoughtfulness might help the staff members grow in confidence. The readiness of a party to be vulnerable to the acts of another party based on the expectation that the other will execute a specific action significant to the trustor, irrespective of the ability to monitor or control that other party, is the definition of trust, according to Mayer et al. (1995) [29]. During the pandemic, such emotional support which includes empathy, care, and encouragement can be especially helpful in helping employees deal with uncertainties like health and mental health issues. Thus, managers who show concern for the welfare of their subordinates might help employees who are emotionally worn out, which builds trust in organizations and leaders [3, 30].

## Gen as a moderator

Organizations are greatly impacted by a multigenerational workforce, particularly managers and leaders who must be able to deal with diversity to function well. In the study of organizational behavior, the significance of generation disparities has increased over time. As a result of this expansion, it has been realized that employee preferences for management and leadership are greatly influenced by generational differences [31]. According to Wey Smola and Sutton (2002), generation is a collection of individuals born within the same period, having similar life experiences, historical events, and social events. As a result, individuals from the same generation may have comparable worldviews and values [32].

Generation is “a group of individuals born and living contemporaneously, who have common knowledge and experiences that affect their thoughts, attitudes, values, beliefs, and behaviors” [33]. Traditionalists (1925–1945), Baby Boomers (1946–1964), Generation X (1965–1980), Generation Y (1981–2000), and Generation Z (born after 2000) were the groups into which the generation was divided. As a result, the age ranges of the following generation are the focus of our study: Baby Boomer nurses who are over 55; X genes who are between 42 and 55 years old; Y genes who are between 25 and 42 years old; and Z genes who are under 25 years old. Research shows that each generation has its characteristics, values, and attitudes towards work [34]. Because of these generational differences, it can be argued that employees from different generations will not respond the same to TM and will evaluate the employment relationship differently.

## Significance of study

Nurses deal with difficulties daily in a fast-paced, high-pressure workplace and are considered a vital component of the healthcare system. Pandemics like COVID-19, long work hours, unpredictable shifts, night shifts, aging, and new medical technologies are some of the challenges they face. These challenges affect the talent management in hospitals, the physical and mental health of the nurse, the psychological contract, and the plan to depart the institution [35–38]. By 2030, the nursing deficit will need to be filled by more than 13 million nurses globally, according to the International Council of Nurses (ICN) 2021 [39]. According to WHO (2022), health systems are negatively impacted by a lack of competent professionals, inadequate recruitment and retention efforts, unappealing working conditions, and limited access to chances for ongoing professional development [40]. Researchers have shown via their studies that a violation of the psychological contract among nurses has detrimental effects on their mental and physical health, as well as their job satisfaction and performance. It can also harm the organization due to a scarcity of nurses [40–43].

Nonetheless, to further enhance the healthcare sector, there must be a rise in the number and caliber of healthcare practitioners, regarded as essential resources in any business due to their significant value addition [41]. The importance of talent management as a strategy for nurses’ psychological contract fulfillment has made it a critical management concern. Nevertheless, a substantial body of research has been done to examine the connection between talent management and psychological contracts, however, other factors such as cordial relation and Gen have not been explored in previous studies. Thus, our study tries to uncover this empirical knowledge gap [44, 45].

### Research hypotheses

H1: Talent management has a significant correlation with psychological contract fulfillment.

H2: Talent management has a positive significant correlation with cordial relations.

H3: Cordial relation has a positive significant correlation with psychological contract fulfillment.

H4: Multigeneration has a positive significant correlation with psychological contract fulfillment.

H5: Multigeneration moderates the relationship between talent management and psychological contract fulfillment.

H6: Cordial relation mediates the relationship between talent management and psychological contract fulfillment.

## Methodology

The methodological structure of this research is developed in four sections: (1) Research design and setting, (2) Participant and sampling, (3) Data collection and variables, and (4) analysis technique. These sections are designed to provide a clear understanding of the research. A detailed description of each section is presented below.

### Research design and setting

This study used a descriptive, cross-sectional, and correlational research design to examine the relationship between variables during a specified time. The cross-sectional approach is known for its efficacy in capturing data at a single point in time and is suitable for exploratory and explanatory research in organizational studies. This study was conducted in three large private hospitals in Alexandria governorate. They provide similar types of healthcare services and are occupied with slightly similar numbers of bed capacity.

### Participant

A non-probability convenience sampling technique was used to recruit the study participants from all departments of the three hospitals to be included in this study. The total number of nurses employed in these three hospitals was 850. Participants who were registered nurses willing to participate, their ages were relevant for Gen classifications, and those who could provide written informed consent were eligible. The exclusion criteria were nurses who had physical or mental illnesses. Based on these exclusion criteria, the final possible population size was 600. The sample size was determined using the Steven K. Thompson equation [46] to calculate the sample size based on the following formula.

$$n=\frac{N \times p(1-p)}{[[N-1 \times (d^{2}\div z^{2})]+p(1-p)]}$$

Where: N = total population, Z = confidence level at 95%, d = error proportion (0.05), p = probability (50%), n = sample size. Thus, the minimum recommended sample size is 314. However, to attain the desired sample size, 400 questionnaires were given to nurses with 100% acceptance to participate in the study; 380 nurses completed the final questionnaires and returned them, which exceeded the required sample size. However, 5 surveys were excluded based on clearance assessment to reach 375 surveys included in the study with 6.25% attrition rate and 93.75% response rate.

### Tools of the study

1. **Talent Management Questionnaire**

This tool was developed by Tiwari and Shrivastava (2013), it consists of 19 items divided into 4 dimensions: Growth and learning opportunities/ talent development (5 items), Compensation and benefits/reward and recognition (4 items), Work environment and policies/ talent policies (7 items), and Management supports leadership support (3 items). The responses were measured using a five-point Likert scale ranging from (1) strongly disagree to (5) strongly agree. The highest score indicates a high level of perceived talent management. Scoring levels start from 19 for a low perception of nurses’ talent management to 95 for a high level of perception [17].

2. **Cordial relations Questionnaire**

This tool was developed by researchers based on the current related literature; Sahoo & Sahoo [7]; Sharma & Sharma [47]; Gill [48] to assess employee perception of cordial relations with their managers. This tool consists of 60 items distributed through 4 dimensions; employee commitment (15 items); employer commitment (23 items); Employee trust (11 items); and employer caring (11 items). The responses were measured on a 5-point Likert scale ranging from (1) strongly disagree to (5) strongly agree. The highest score indicates a high nurse perception of cordial relations. The scoring level ranges from 60 for a low level to 300 for a high level of cordial relation [7, 45, 46]. Please see the supplementary file.

3. **Psychological contract fulfilment questionnaire (PCF)**

This tool was developed by Robinson and Morrison (1995). It consists of 4 items to assess the nurses’ perception of their psychological conduct fulfillment focusing on the individual’s beliefs in terms of an exchange agreement between the individual and his or her organization and comprising the employee’s feelings about the employer’s obligation regarding pay and support. The response was measured on a 5-point Likert scale ranging from (1) strongly disagree to (5) strongly agree. The highest score indicates a high perception of psychological contract fulfillment. Scoring levels range from 4 for low perception to 20 for high perception of psychological fulfillment [49].

In addition, **personal and professional-work related data** of the study subject includes questions related to multi-generation level, gender, level of education, and marital status.

### Validity and reliability

The three questionnaires were tested for content validity by a panel of experts in the related field, after that the content validity indices were measured and revealed that the study tools were valid with content validity indices equal 0.90, 0.87, and 0.83 for the talent management questionnaire, cordial relations questionnaire, and psychological contract fulfillment questionnaire, respectively. Also, the three tools were tested for their reliability using Cronbach’s alpha coefficient, the results proved that the three tools were reliable with a coefficient value of 0.716, 0.816, and 0.795 for the talent management questionnaire, cordial relations questionnaire, and psychological contract fulfillment questionnaire respectively. In addition, a pilot study was carried out on 37 nurses with no change in the final tool.

### Construct validity

Both exploratory factor analysis (EFA) and confirmatory factor analysis (CFA) were used to assess cordial relations questionnaire construct validity. The exploratory factor analysis (EFA) was done to determine whether the component structure is consistent or varies among the examined nurses to verify the construct validity of the produced tool in the study setting. The EFA was applied using Principal Component Analysis and Varimax with Kaiser Normalization with Kaiser-Meyer-Olkin (KMO) test to pinpoint the fundamental items that define dimensions of the developed cordial relations tool. High values close to 1.0 of the KMO measure of sampling adequacy indicate that factor analysis may be useful with data; however, if the value is less than 0.50, the results won’t be useful. In our analysis, the KMO statistic for the data was 0.89, this indicated suitability for factor analysis test of sample adequacy for cordial relations indicating the data were highly appropriate for factor analysis and showed a high level of item-level common variance for each scale [50].

We conducted the CFA using four factors in full Structural Equation Modeling (SEM); the full model evaluates how well the factor structure of the cordial relations tool suited the data. The models X2, Comparative Fit Index (CFI), Incremental Fit Index (IFI), and Root Mean Square Error of Approximation (RMSEA) were used to judge whether the model fits the data. The recommended Chi-squared p-value should be > 0.05 (insignificant); however, it is quite sensitive to sample size. RMSEA of less than 0.05 is considered well, 0.05 to 0.08 is considered acceptable, 0.08 to 0.1 is considered marginal, and more than 0.1 is considered poor. The CFI and IFI values should be more than 0.96 [51]. The results of the CFA reveal a CFI = 1.000, IFI = IFI = 1.002 indicating perfect fit, and the RMESA = 0.06 indicating acceptable fit. Therefore, we can conclude that the suggested model fits the data reasonably well. See supplementary file.

### Pilot study

A small-scale test was conducted with 20 nurses representing 10% of the study population. The pilot study’s main objectives were to evaluate the study tools’ usability and clarity, spot any possible problems that might be encountered while gathering data and estimate the time needed to complete the questionnaire. It is important to note that the pilot study participants did not comprise the main study sample. The findings from the pilot research demonstrated that no modifications were needed, and the tools were simple to use and understand.

### Data collection

After ethical approval was sought from the Ethics Research Committee (ERC) for the faculty of nursing at Alexandria University IRB00013620 (9/19/2025) and before the beginning of data collection, formal permission was requested from the hospital’s administration. The nursing supervisors of the relevant facilities were also contacted to set up the day of the questionnaire administration. Prospective participants were contacted and recruited for the data collection with the help of the nurse managers and ward in charge. Each nurse completed the questions in about 20 min. The researchers personally administered the scales from May to August of 2023. The study’s participants voluntarily agreed to participate and were made aware that they might withdraw from the study at any time while the data was being collected. Possible risks and benefits of the study were also explained, after which written consent was sought from participants.

Confidentiality and anonymity were also upheld during the whole investigation. After participants finished the questionnaires, the researchers gathered the completed responses. The cleaning procedure involved excluding the responses from participants who did not fulfill the study’s specified requirements or showed indications of inattentive responding—such as incomplete survey responses, and recurring answer patterns. Based on these 5 surveys were removed from the collected data.

### Data quality management

Throughout the research cycle, the researchers employ techniques such as data profiling, cleansing, validation, and monitoring to enhance the general quality of the data and improve data correctness and integrity, anomalies, mistakes, redundancies, and inconsistencies must be found and addressed.

### Statistical analysis

The collected data was revised, coded, and fed into the statistical software program *SPSS*, version 25. The mean score and standard deviation were used to describe the scaled data, while the frequencies and percentages were used to describe the categorical data. Cronbach’s alpha was used to measure the internal consistency of the instruments. Pearson correlation coefficient analysis (r) was used to test the relationship between talent management, cordial relation, psychological fulfillment, and Gen. Path analysis with structural equation modeling was done with SPSS Amos to confirm that the measurement model had an adequate fit. All statistical analyses were performed using 2-tailed tests and an α-error of *p* ≤ 0.05. A significant level was considered at *p* < 0.001. The effect of each variable is estimated, and all results were significant at 0.05. The model fit indices included the evaluation of; χ2/df = Chi-Square/degree of freedom, CFI = Comparative fit index; RMSEA = Root Mean Square Error of Approximation; GFI = Goodness of Fit Index; AGFI = Adjusted Goodness of Fit. (See Table 1)

**Table 1:** The model fit indices

Fit parameter	Good fit	Acceptable fit
χ2 p-value χ2/df	0 ≤ χ2 ≤ 2df 0.05 < *p* ≤ 1.00 0 ≤ χ2/df ≤ 2	2df < χ2 ≤ 3df 0.01 ≤ *p* ≤ 0.05 2 < χ2/df ≤ 3
RMSEA	0 ≤ RMSEA ≤ 0.05	0.05 < RMSEA ≤ 0.1
CFI	0.97 ≤ CFI ≤ 1.00	0.95 ≤ CFI < 0.97
GFI	0.95 ≤ GFI ≤ 1.00	0.90 ≤ GFI < 0.95
AGFI	0.90 ≤ AGFI ≤ 1.00	0.85 ≤ AGFI < 0.90

**Table 2:** Frequency distribution of the studied nurses according to their work-related data

Demographic and work-related characteristics		No	%
Gender	Male	10	2.7
	Female	365	97.3
Generation	B	3	0.8
	X	20	5.3
	M	131	34.9
	Z	221	58.9
Marital status	Single	263	70.1
	Married	94	25.1
	Divorced	16	4.3
	Widow	2	0.6
Educational characteristics	SNSD	87	23.2
	THID	163	43.5
	BSC	125	33.2

**Table 3:** Mean and standard deviation of talent management with its’ related dimensions and psychological contract fulfillment from nurses’ perspective

Study variables	Low		Moderate		High		Mean ± SD
	No	%	No	%	No	%	
Growth and learning dimension	105	28.0	151	40.3	119	31.7	16.00 ± 5.43
Compensation dimension	265	70.7	100	26.7	10	2.7	7.95 ± 3.55
Talent policy dimension	139	37.1	66	17.6	170	45.3	18.20 ± 7.92
Leadership dimension	248	66.1	99	26.4	28	7.5	6.75 ± 3.50
**Total talent management**	**125**	**33.3**	**174**	**46.4**	**76**	**20.3**	**48.91 ± 18.15**
**Psychological contract fulfillment**	267	71.2	64	17.1	44	11.7	8.89 ± 3.93

The study was authorized by the Ethics of Research Committee of the Faculty of Nursing Alexandria University IRB00013620 (9/4/2023). Following approval from the hospital’s administration and before embarking on data collection, written informed consent was obtained from participants after explaining the aim of the study and assuring the confidentiality of the gathered data, securing anonymity, and respecting privacy. Participants were informed that they could opt out of the study.

## Results

Table 2 Reveals that most nurses were female (97.3%). Regarding the generation, 58.9% are related to the Z Gen, followed by Millenium 34.9%, and only 0.8% of the studied nurses in the Baby Boomer generation. According to their marital status, one quarter were married (25.1%) and 70.1% were single. According to educational level, it is apparent that two-thirds of studied nurses (43.5%, 23.2%) had a Technical Health Nursing Institute Diploma and a Secondary Nursing School Diploma respectively, while 33.2% had a BSc.

Table 3 demonstrates that nearly half (46.4%) of nurses perceived a moderate level of talent management with a mean score of (48.91 ± 18.15) this is represented by 70.7%, 66.1% of nurses perceived a low level of compensation and leadership dimension with a mean score (7.95 ± 3.55, 6.75 ± 3.50) respectively. Furthermore, 40.3% perceived a moderate growth and learning dimension with a mean score (16.00 ± 5.43). Otherwise, 45.3% perceived a high talent policy dimension with a mean score (18.20 ± 7.92). Concerning psychological contract fulfillment, this table reflects that 71.2% of nurses perceived low psychological contract fulfillment with a mean score (8.89 ± 3.93).

Table 4 Declares that most nurses (87.5%) perceived a moderate level of cordial relations with a mean score of 185.11 ± 27.02, and this can be explained as 91.2% of nurses perceived low employer commitment with a mean score (38.03 ± 11.15). Also, 61.9% perceived moderate employee trust with a mean score (25.73 ± 5.15). However, 93.3% and 93.1% of nurses perceived high employee commitment and employer caring with mean scores of 72.61 ± 13.24, and 48.73 ± 3.48 respectively.

**Table 4:** Mean and standard deviation of cordial relation from nurses’ perspective

Cordial relation dimensions	Low		Moderate		High		Mean ± SD
	No	%	No	%	No	%	
Employer commitment	342	91.2	33	8.8	0	0%	38.03 ± 11.15
Employee commitment	0	0%	25	6.7	350	93.3	72.61 ± 13.24
Employee trust	143	38.1	232	61.9	0	0%	25.73 ± 5.15
Employer caring	0	0%	26	6.9	349	93.1	48.73 ± 3.48
**Total cordial relations**	0	0%	328	87.5	47	12.5	185.11 ± 27.02

**Table 5:** The correlation matrix between talent management and psychological fulfillment contract, generation, and cordial relation

		Generation	Talent management	Psychological contract
Talent management	**r** **P**	0.327^**^ 0.000	1	
Psychological contract fulfillment	**r** **P**	0.199^**^ 0.000	0.854^**^ 0.000	1
Cordial relations	**r** **P**	0.306** 0.000	0.919^**^ 0.000	807^**^ 0.000

**Correlation is significant at the 0.01 level (2-tailed)

Table 5 Reflects that there was a highly statistically significant positive correlation between talent management and generation, psychological contract fulfillment, and cordial relation where (*r* = 0.327, *p* = 0.000, *r* = 0.854, *p* = 0.000, *r* = 0.919, *p* = 0.000). Also, there was a highly statistically significant positive correlation between generation and psychological contract fulfillment and cordial relations where (*r* = 0.1999, *p* = 0.000, *r* = 0.306, *p* = 0.000).

**Table 6:** Mediation-moderation model of cordial relation, generation, talent management, and psychological contract fulfillment

Study variables			Standardized beta	Unstandardized beta	S.E.	C.*R*.	*P*
Talent management	⟶	Cordial relation	1.017	0.559	0.013	42.761	***
Cordial relation	⟶	Employer commitment	0.895	1.000			
Cordial relation	⟶	Employee commitment	0.706	0.936	0.052	17.843	***
Cordial relation	⟶	Employee trust	0.949	0.490	0.015	33.026	***
Cordial relation	⟶	Psychological contract fulfillment	0.961	0.375	0.090	4.193	***
Gen *talent management	⟶	Psychological contract fulfillment	− 0.080	− 0.004	0.003	-1.371	0.170
Gen	⟶	Psychological contract fulfillment	− 0.313	-1.919	0.236	-8.128	***
Talent management	⟶	Psychological contract fulfillment	0.212	0.046	0.052	0.876	0.038
Cordial relation	⟶	Employer caring	− 0.011	− 0.004	0.017	− 0.221	0.825

Model fit indices: X^2^ = 323.428 = CMIN = 109.391, p value = 0.000; GFI = 0.927, AGFI = 0.813; CFI = 0.977; REMSA = 0.135

**Fig. 2:**
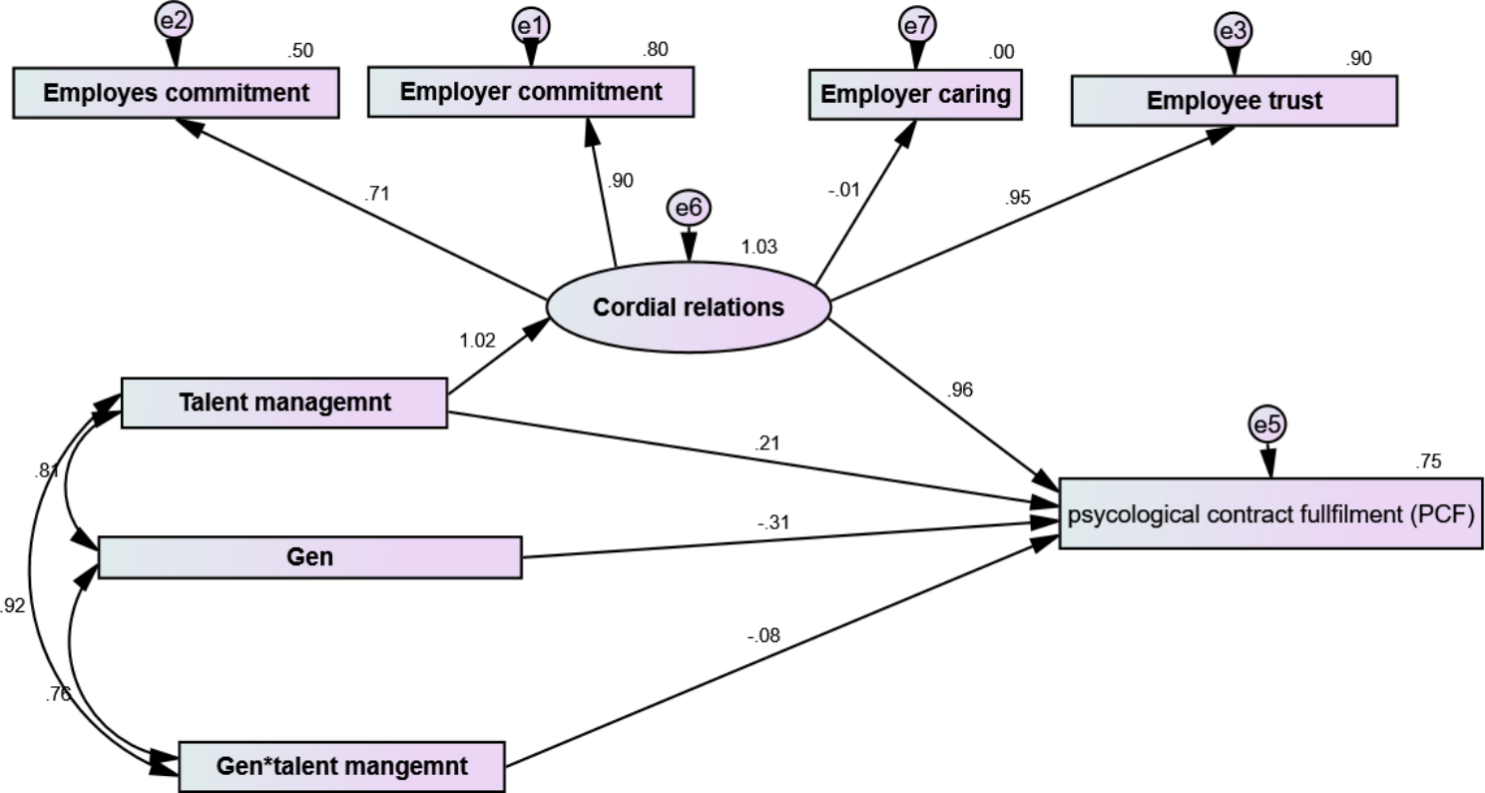
Mediation-moderation Model of Cordial Relation, Generation, Talent Management, and Psychological Contract Fulfillment

We examined a structural model with 50 rotations at 95% confidence to test the hypotheses and used standardized beta to interpret this model. Table 6; Fig. 2 illustrate that a positive prediction of talent management to PCF among nurses (*β* = 0.212, *p* = 0.038), confirming H1. Additionally, cordial relations predict PCF (β = 0.961, *P* < 0.001). While GEN negatively predicted PCF (*β* = -313, *p* = 0.003). These results support the two hypotheses of the study H2, and H3.

The structural equation modeling (SEM) results indicate that after introducing cordial relation as a mediating variable, talent management predict increase in PCF and the total effect was = 1.189, Direct effect of talent management = 0.212, and its indirect effect = 0.977. Additionally, by computation an interaction between Gen and talent management, the moderating influence of Gen on psychological contract fulfillment and talent management was evaluated. Gen had a substantial detrimental impact on PCF, as we observed (ß = -0.313, *p* = 0.038). However, It is positively correlated with talent management, (ß =0.810, *p* = 0.002). Conversely, the interactional term (Gen *talent management) was found to have significant effects (ß = − 0.20, *p* = 0.000), indicating moderation as it modifies the direction of the relationship between psychological fulfillment and talent management and accounts for 38% of the variance effect. that indicate baby boomer had predict PCF and the nurse manger should develop strategy to enhance other gens PCF.

## Discussion

Healthcare organizations should use talent management, a field that has developed over the past 20 years and gained greater attention in the private sector but has not been given the same consideration in the industry, to build a more sustainable staff. Since talent management is becoming the most essential managerial concern in this extremely dynamic and frequently uncertain market environment of the twenty-first century, it is becoming recognized as a vital success component within businesses. Furthermore, it is one of the key processes that favorably affect innovative work behavior, work engagement, career orientation, job satisfaction, and employee retention in businesses [52, 53].

In our study, nurses perceived a moderate level of talent management. This result may be related to nurses perceived a moderate level of growth and learning and more than two-thirds of them perceived low compensation and leadership. Otherwise, one-third of them perceived a high talent policy. This result is supported by Chelan et al. 2022; Alferjany et al., 2022 whose study revealed moderate talent management [54, 55]. However, this contrasted with the results of Dzimbiri and Molefakgotla, 2021; Lawal et al., 2022 whose study concluded a low level of talent management and reported that 70% of respondents rated their talent management practices as poor or very poor [56, 57]. Otherwise, Yener et al., 2017; Mahfoozi et al., 2018, Kheirkhah et al., 2016; Pomaranik et al. 2023 in their study reported a higher level of talent management [58–61]. These contradictory results offer clear insights and challenges for the hospital administrator to look forward and redesign their strategy to enhance talent management. These can be achieved through implementing a training program for their managers. Additionally, education has a great role in incorporating talent management into its curriculum. This result heightened the way for the hospital administrator to develop a short-term strategy to attract young students to the organization by offering internships after they complete their studies.

From the standpoint of the previously explained theories, the talent management process does not matter to the talented employee in two ways. Firstly, being added to a talent pool signifies that the employer is trying to uphold the terms of the psychological contract (PC). Due to its significance in comprehending the exchange relationship between employers and employees, PC has drawn more attention [20]. In our study, nurses reported a low level of psychological contract fulfillment. This may be explained in the light of the discussed theories on a norm of reciprocity as the employer fulfills promised obligations nurses are more likely to increase feelings of obligations to reciprocate and it is used to comprehend the nature of the working relationships and involves mutual the responsibilities that employers and their employees have to one another. This result parallels van et al. (2020) who reported that psychological contract fulfillment decreased due to organizations failing to keep their promises [62]. However, it contradicted Varma and Chavan (2020); and Yu (2022) whose study revealed a moderate level of psychological contract fulfillment [63].

Cordial relations are based on justice, trust, and respect between each other which provide the best outcomes for the company. One goal of industrial relations is to foster friendly ties with employees; companies want to be pleasant and approachable in their interactions with staff. A supervisor-subordinate relationship of the highest caliber is excellent for employees in an organization [56, 64]. Despite this, our study revealed that nurses perceived a moderate level of cordial relation, and this can be explained in 91.2% of nurses perceived low employer commitment, and moderate employee trust. However, 93.3% and 93.1% of nurses perceived high employee commitment and employer caring. This contradicted Haridas 2022 whose study revealed a low mean score of cordial relation [64].

Based on the previous, the result of our study revealed a highly statistically significant relationship between generation, talent management, psychological contract fulfillment, and cordial relations. Also, talent management positively predicts psychological contract fulfillment by 0.212. Furthermore, talent management could positively predict cordial relations by β = 1.017. That means cordial relations partially mediated the relationship between talent management and psychological contract fulfillment, and the total mediating effect was Total effect = 1.189, the direct impact of talent management = 0.212, and its indirect effect Indirect = 0.977. Also, Gen negatively predicts the psychological fulfillment contract (*β* = -0.13, *p* = 0.003). While, it has a positive association with talent management (*β* = 0.810, *p* = 0.002) which indicates moderation as it changes the direction of the relationship between talent management and psychological fulfillment and is responsible for the variance effect by 38%.

This result is consistent with Goswami, 2021, Raheem & Khan (2019), Sandeepanie et al., (2023), Van den Heuvel (2014) and Farr & Ringseis, (2002) who reported a positive relationship between talent management in organization, psychological contracts, and cordial relation among employees [2, 3, 65–67]. However, this contradicted Plessis (2010) whose study revealed that the Generation acts as a positive moderating variable between psychological contract fulfillment and talent management [68]. They depicted that Baby Boomers (older employees) tend to have more stable psychological contracts because they have a longer tenure with one organization, are more experienced, and have a more positive view towards life and events because they are reaching the end of their careers. Younger employees will have enough opportunities to work in other organizations when the employment relationship might end and therefore are less concerned with the way their relationship ends. Also, generation Y is likely to have talent management practices, generation Y’s view of the world has been strongly influenced by an electronic world, information overload, and overzealous parents [57, 62, 69].

Additionally, Sahoo, & Sahoo [7], and Mehta et al., (2024) were consistent with our results and proved that cordial relation acts as a mediating variable and enhances the relationship between talent management and psychological contract and clarified that there is alignment between employee-employer relation in the form of good cordial relation, trust, caring, and commitment with talent management strategy of the organization which had a good impact on psychological contract fulfillment leads to motivated, loyal and high-performing employees and facilitates them to achieve the optimum results for their organization [7, 70]. Therefore, this study theoretically strengthens and reinforces the developed moderating-mediating model between talent management, nurses’ psychological contract fulfillment, Gen as a moderating component, and cordial relation as a moderating factor.

Thus, these findings suggest that administrators should consider that talent management techniques are strategically aligned with the organizational goals because talent management initiatives maximize the employees’ psychological contracts. Additionally, they must adopt inclusive policies that meet the requirements of all age groups or generational differences. The inclusive talent mindset is predicated on the idea that all current employees, regardless of the abilities they possess, are deemed talented. Similarly, every generation possesses a distinct set of abilities and skills that need to be utilized to optimize organizational performance. Therefore, the implementation of proper talent management policies, processes, and programs significantly impacts staff psychological fulfillment and enhances their cordial relation with their VUCA healthcare organization.

## Conclusion

This research constructs a moderated mediation model between the study variables confirming the positive influence of talent management on nurses’ psychological fulfillment and demonstrating the mediating effect of cordial relations and the moderating effect of genes in this relationship. GEN negatively predicted psychological contract fulfillment where baby boomers predicted more psychological contract fulfillment than Z gen. Moreover, there is a significant effect of Gen on talent management which indicates moderation. Furthermore, talent management could positively predict cordial relations and psychological contract fulfillment, and cordial relations partially mediated the relationship between talent management and psychological contract fulfillment. Thus, Nurse Mangers should be aware of and implement effective talent management strategies with respecting gen difference, and tailored strategies for fostering nurses’ cordial relations and psychological contract fulfillment as open discussion of individual concerns and implementing the open-door policy to deal with the VUCA challenging healthcare environment.

### Limitations of the study

Although this study constructed a moderated mediation model between talents management, nurses’ psychological contract fulfillment, Gen as a moderating factor, and cordial relation as a mediating factor. Some limitations provide opportunities for further studies in the area. First, a cross-sectional data collection method from three hospitals relying on a non-probability convenience sampling technique may restrict generalizability. Second, even though this study employed the SEM approach, the cross-sectional study design makes it impossible to demonstrate causality and limits the evaluation of the influence of study factors. Finally, the data was collected through self-report measures that may be sensitive to subjectivity and response bias. To address these limitations, objective metrics will be required in the future through observational, longitudinal, qualitative, empirical, interview, experiment, and multi-site research.

### Implications

This study adds to the corpus of information, research, and practice and has applications for staff nurses, nurse supervisors, and hospital human resources administration procedures. Enthusiastic researchers can contribute to empirical studies to discover mediating and moderating variables. Managers and practitioners can use these study results to build an efficient organizational model, foster positive employee-employer interactions, and develop a culture of harmony and trust between organization holders. Additionally, the present study enhances the bond between employers and employees while aiding organizations in maintaining flexibility and achieving long-term sustainability. These findings motivate managers and leaders to invest time, funds, effort, and other resources in building a successful talent management system. They can do this by creating strategies and policies for sustaining improved talent management practices and fostering amicable relationships between nurses from various generations. Also, managers should be careful about what promises and expectations they create with talented employees of different generations. Also, they should communicate effectively with their high-potential employees about the promises, business situations, and other factors that may influence or limit their ability to fulfill their promises to their talented employees. To achieve this, a clear and compelling strategy must be developed to attract, engage, and retain staff across the different generations. By becoming more aware of the needs and preferences of their diverse workforce, organizations can develop suitable talent management strategies.

## Electronic supplementary material

Below is the link to the electronic supplementary material.

Supplementary Material 1

## Data Availability

The datasets used and/or analyzed during the current study available from the corresponding author on reasonable request.

## Declarations

### Content of the manuscript

We confirm that the content of the manuscript has not been published or submitted for publication elsewhere.

### Approval confirmation

All authors have approved the manuscript for submission.

## Abbreviations

ANOVA: Analysis of variance
SPSS: Statistical package of social science
SD: Standard deviation
TM: Talent Management
PCF: Psychological Contract Fulfillment
SET: Social Exchange Theory
SNSD: Secondary Nursing School Diploma
THID: Technical Health Nursing Institute Diploma
B: Baby boomer
X: Gen X
M: Millenium
Z: Gen Z
