# Supplementary material for: Talent management in volatility, uncertainty, complexity, and ambiguity (VUCA) health environment, nurses’ psychological contract fulfillment, cordial relation and generation: moderation-mediation model
Source: BMC Nurs. 2024 Dec 3;23:883. doi: 10.1186/s12912-024-02506-7 (PMC11613867; doi:10.1186/s12912-024-02506-7)

**Talent Management in** **Volatility, Uncertainty, Complexity, and Ambiguity (VUCA) Health Environment, Nurses’ Psychological Contract Fulfillment, Cordial Relation and Generation: Moderation-Mediation Model**

Supplementary file:

Exploratory factor analysis


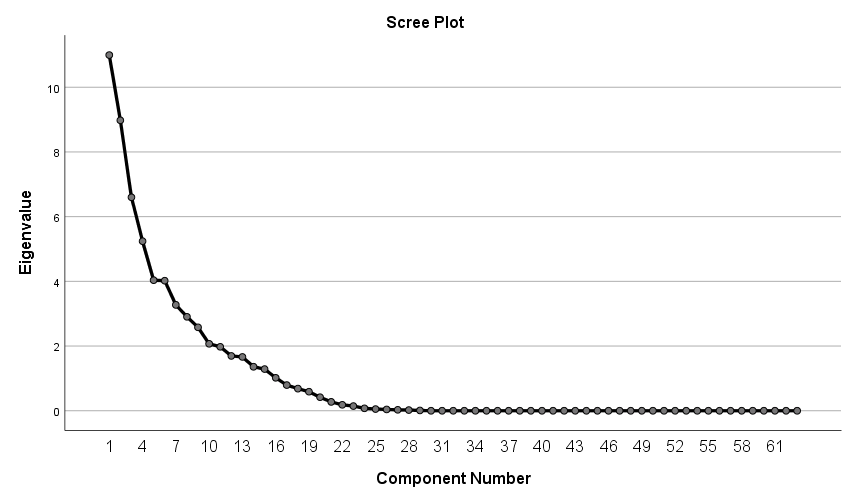


**Figure (3) Component factor loading with varimax (1), and correlation coefficient at 0.30 of the developed cordial relations**

**Table 7Rotated Component Matrix**

|  | Component | | | |
| --- | --- | --- | --- | --- |
|  | 1 | 2 | 3 | 4 |
| 1. Concern for my personal welfare | .491 |  |  |  |
| 1. Be responsive to employee concerns |  |  | -.787 |  |
| 1. Make decisions with my | .470 |  |  |  |
| 1. Concern for my long-term well-being | .633 |  |  |  |
| 1. Limited involvement in the organization | -.624 |  |  |  |
| 1. Support me to attain higher levels | .644 |  |  |  |
| 1. Help me respond to each greater | .733 |  |  |  |
| 1. Support me in meeting higher goals | .713 |  |  |  |
| 1. Developmental opportunities within this firm | .749 |  |  |  |
| 1. Advancement within the firm | .703 |  |  |  |
| 1. Opportunities for promotion | .735 |  |  |  |
| 1. Help me develop extremely marketable skills | .701 |  |  |  |
| 1. Job assignments that enhance | .786 |  |  |  |
| 1. Potential job opportunities outside | .583 |  |  |  |
| 1. I have no future obligations(r) |  |  |  |  |
| 1. Leave at any time I choose(r) |  |  |  | .795 |
| 1. I have much fewer commitments(r) |  | .718 |  |  |
| 1. Make personal sacrifices for this organization |  |  | .614 |  |
| 1. Take this organization's concerns personally ) |  | -.451 |  |  |
| 1. Protect this organization's image |  |  |  | .728 |
| 1. Commit myself personally |  |  |  | .571 |
| 1. Seek out assignments that enhance the value |  |  |  | .486 |
| 1. Build skills to increase my value in this organization |  |  |  | .874 |
| 1. Make myself increasingly valuable to this employer |  |  |  | .609 |
| 1. Actively seek internal opportunities |  |  |  | .371 |
| 1. Accept increasingly challenging performance standards |  |  |  | .675 |
| 1. Take personal responsibility |  |  |  | .837 |
| 1. Continually exceed my formal accomplishments |  |  |  | .680 |
| 1. Build contacts outside firm | .375 |  |  |  |
| 1. Increase my visibility |  | .613 |  |  |
| 1. Building skills to increase future employment |  | .666 |  |  |
| 1. Seek out assignments that enhance my employability |  |  |  | .579 |
| 1. Withholds information from me(r) |  |  |  | .394 |
| 1. Doesn't trust me(r) |  |  | .339 |  |
| 1. Introduces changes without involving me (r) |  |  | -.607 |  |
| 1. Doesn't share important information with me (r) |  |  |  | .441 |
| 1. Difficult to predict future direction of its relations with me (r) |  |  |  | -.442 |
| 1. An uncertain future regarding its relations with me (r) | -.688 |  |  |  |
| 1. Uncertainty regarding its commitment to employees (r) |  | -.700 |  |  |
| 1. Uncertainty regarding its commitment to me (r) |  | -.628 |  |  |
| 1. Demands more from me while giving me less in return (r) |  | -.397 |  |  |
| 1. Decreasing benefits over the next few years (r) |  |  | -.546 |  |
| 1. Stagnant or reduced wages the longer I work here (r) |  |  | -.500 |  |
| 1. More and more work for less pay (r) | -.488 |  |  |  |
| 1. Contracts that create employment opportunities elsewhere | .577 |  |  |  |
| 1. Perform only required tasks(r) |  | .504 |  |  |
| 1. Do only what I am paid to do( r) |  | .797 |  |  |
| 1. Fulfill a limited number of responsibilities(r) |  | .615 |  |  |
| 1. Only perform specific duties(r) |  | .530 |  |  |
| 1. Quit whenever I want(r) |  | -.564 |  |  |
| 1. I cannot believe what this employer tells me (r) |  |  | -.375 |  |
| 1. I have no trust in this employer (r) |  | .748 |  |  |
| 1. Inconsistency exists between what this employer says and does (r) | .549 |  |  |  |
| 1. I'm getting less pay for more work (r) |  | .371 |  |  |
| 1. I'm doing more for less (r) |  |  | .404 |  |
| 1. I expect increasing demands from this employer for little return (r) |  |  | .683 |  |
| 1. It's difficult to predict the future of this relationship (r) |  |  | .752 |  |
| 1. I cannot anticipate what my future relationship (r) |  |  | .566 |  |
| 1. It's difficult to anticipate my future commitments (r) |  |  | .574 |  |
| 1. My commitments to this employer are uncertain (r) |  |  | .665 |  |
| Extraction Method: Principal Component Analysis.  Rotation Method: Varimax with Kaiser Normalization. | | | | |
| a. Rotation converged in 8 iterations. | | | | |

**Confirmatory factor analysis to the developed Cordial Relations questionnaire**

**Table 7 Standardized Regression Weights**

|  |  |  | Estimate | S.E. | C.R. | P | Label |
| --- | --- | --- | --- | --- | --- | --- | --- |
| Employee commitment | <--- | Cordial relations | .948 |  |  |  |  |
| Employer commitment | <--- | Cordial relations | .743 | .052 | 17.876 | *** | par_1 |
| Employee trust | <--- | Cordial relations | .889 | .019 | 23.318 | *** | par_2 |
| Employer caring | <--- | Cordial relations | -.026 | .018 | -.478 | .633 | par_3 |

**Figure (4) of CFA of cordial relations questionnaire**


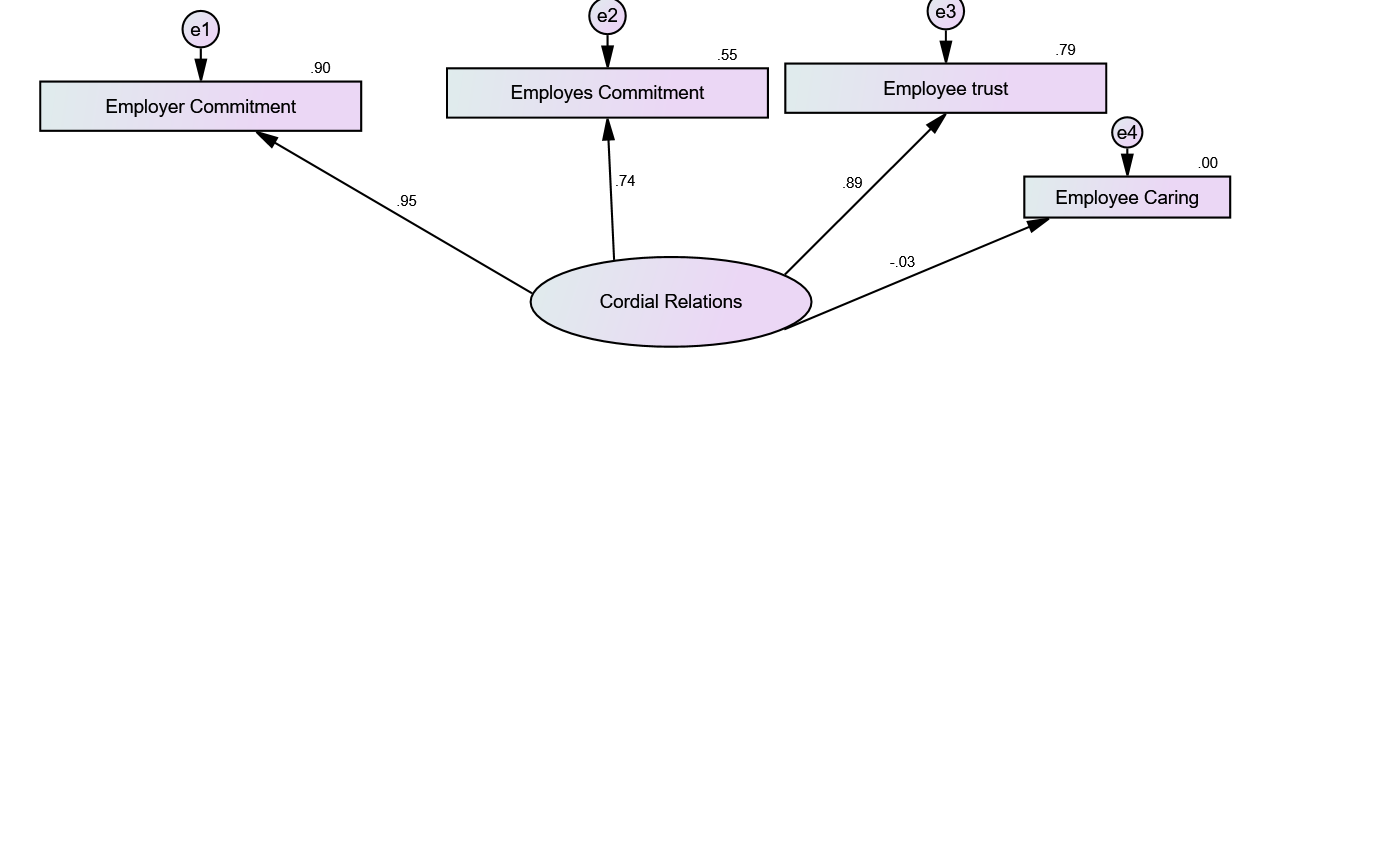

Supplement: Supplementary file 1 — Supplementary Material 1 [file 12912_2024_2506_MOESM1_ESM.docx]
